# Experimental Research on Deep-And-Narrow Micromilled Grooves Using a Self-Fabricated PCD Micro-Cutter

**DOI:** 10.3390/mi12101170

**Published:** 2021-09-29

**Authors:** Jinjin Han, Rui Ma, Xiuqing Hao, Linglei Kong, Ni Chen, Liang Li, Ning He

**Affiliations:** 1 College of Mechanical Engineering, Jiangsu University of Technology, Changzhou 213001, China; hanjinjin2021@126.com (J.H.); nuaamarui@163.com (R.M.); konglingl@nuaa.edu.cn (L.K.); 2 College of Mechanical and Electrical Engineering, Nanjing University of Aeronautics & Astronautics, Nanjing 210016, China; ni.chen@nuaa.edu.cn (N.C.); liliang@nuaa.edu.cn (L.L.); drnhe@nuaa.edu.cn (N.H.)

**Keywords:** hybrid process, PCD micromilling cutter, large-aspect-ratio, surface quality, tool wear

## Abstract

Deep-and-narrow micro-grooves are the common functional structures of miniature parts. The fabrication of the micromilled grooves with high quality and accuracy is the essential guarantee of the causative performance for these miniature parts, and micromilling is the most versatile process to machine such micro-grooves. However, micromilling technology is a highly tool-dependent process, and the commercial carbide micromilling cutter has shown obvious deficiencies in terms of rapid tool wear and inferior machined quality during the machining process. In this paper, a polycrystalline diamond (PCD) micromilling cutter with a large-aspect-ratio (LAR) was designed and prepared by the self-proposed hybrid fabrication method of laser and precision grinding. Micromilling experiments on oxygen-free copper were conducted, and the carbide micromilling cutter was selected in the comparative experiments. The variations of milling forces and specific energy were analyzed through the parameter experiments. Then, the surface quality, machined accuracy and tool wear were further investigated. Results showed that the PCD micromilling cutter with an aspect ratio of 3.25 was successfully manufactured by the proposed hybrid method. The self-fabricated PCD micromilling cutter presented remarkable superiority in terms of the surface quality, machined accuracy, and tool wear when preparing deep-and-narrow micro-grooves. Finally, a satisfactory micromilled groove with an aspect ratio of 2.5 was achieved with the self-fabricated LAR PCD cutter under the optimized conditions.

## 1. Introduction

In recent years, the requirement for and application of microdevices with complex structures have been rapidly increasing in the national defense and civil fields. The deep-and-narrow microgrooved structure has become a research hotspot in mechanical field because it can reduce workpiece weight and material consumption under the premise of ensuring its stiffness and strength. Deep-and-narrow micro-groove is a critical functional structure of the Terahertz (THz) slow-wave structure [1,2], microforming die and micro-heat exchanger [3], etc. The common characteristics of these structures are [4,5]: (1) material diversity (including metal, ceramics, and composite materials); (2) complex geometric structure with large-aspect-ratio and small scale; (3) high machined quality and consistency; and (4) strict machining accuracy and high sidewall perpendicularity, etc. The service performance of miniature parts is dominated by the machined quality and precision of this kind of microstructure, but the fabrication difficulty is still a problem.

At present, the alternative processing technologies for the deep-and-narrow micro-grooved structures primarily include electromachining technology (EDM and ECM), LIGA/UV-LIGA, grinding, micromilling, etc. In contrast, micromilling is one of the most reliable micromanufacturing methods for these micro-grooved structures, because of the advantages of various materials, high efficiency, controllable machined accuracy and low-cost [6]. Bang et al. [7] fabricated a group of 200-μm-width wall microstructures with an aspect ratio greater than three on a 5-axis micromilling machine. Llanos et al. [8] researched the surface quality, milling strategies and tool paths of thin walls by the commercial carbide micromilling cutters. Parameter optimization experiments were conducted and the machining capability was verified by machining 750-μm-deep and 50-μm-thick thin walls on CuZn36Pb3 brass. Two-fluted coated carbide microcutters were used for machining the Beryllium bronze, and a 14-μm-thick thin-walled structure was obtained by adopting an auxiliary support [9]. A 16-blade aluminum alloy 1100 micro-impeller with average blade thickness of 11.96 μm was manufactured by 200-μm-diameter two-fluted carbide micromilling tools [10]. However, the commercial coated carbide cutter with large-aspect-ratio has obvious shortcomings in machining the softer metal microstructures, for instance, serious coating-shedding, premature tool failure and uneven milling phenomenon, which further deteriorates the machined surface quality. Meanwhile, frequent tool replacement not only increases the tool consumption, but also introduces unnecessary repeated positioning error and tool clamping error to the manufacturing process [11,12].

Microcutter technology is one of the most critical elements to the extensive application of micromilling technology, the machined surface quality and machined dimensional accuracy of microstructures is determined by the micromilling cutter [13]. Hence, the preparation of super-hard diamond cutters with high aspect ratio and long service life is the critical factor to solve the above difficulties. Due to the high hardness, high compressive strength and wear-resistance, polycrystalline diamond (PCD) compounded by microdiamond powders with metallic or ceramic binders becomes the most popular and promising diamond material for micromilling cutters and shows superior performance in micromilling [6]. For instance, Nakamoto et al. [14] machined tungsten carbide material with a PCD micromilling cutter, micro-grooves processed with a depth-to-width ratio of 5 were obtained and the peak–valley surface roughness was below 40 nm. Precision fabrication of PCD micromilling cutters is a very challenging task, because the feature dimensions and rigidity are very limited. The available preparation techniques for PCD micromilling cutters mainly include the pulse laser [15], conventional grinding [16,17], micro electric discharge machining (micro-EDM) [18] and multiple process manufacturing technology [19]. The diamond-wheel grinding and polishing was used to prepare 20-edge PCD micromilling cutters by Suzuki et al. [20]. A PCD end-mill with a designed hexagonal geometry was fabricated using WEDM [21]. The surface roughness and cutting-edge radius achieved were about 0.11 μm and 1.8 μm, respectively. The femtosecond laser was further utilized to fabricate a binderless polycrystalline diamond (BLPCD) cutter [22]. The cutting-edge radius and surface roughness obtained were 0.8 μm and 22 nm, respectively, and no graphite residue was found on the tool surfaces.

Single conventional grinding was the primary fabrication technique for the non-ultra-hard micromilling cutters before 2010 due to the low efficiency, intensive wheel wear, and insufficient accuracy. Whereas, for the ultra-hard micromilling cutters, single conventional grinding is no longer recommended, nonconventional technologies such as wire electro discharge machining and pulse laser ablation have become more popular. An additional postprocessing operation is needed due to the recast generated in micro-EDM, which makes the preparation process of micromilling cutters more complex. Limitations of using the pulse laser are the expensive price for picosecond and femtosecond lasers and substantial preparation efforts and repeated parametric trials for different tool material [19]. With constant advances in the fabrication technology, a new multiple-process fabrication (MPF) method is also proposed. Based on the combination of a picosecond laser and FIB, a cemented carbide ball micro milling cutter with the cutting edge radius of less than 1 μm was fabricated [23]. By combining a nanosecond laser and grinding, a CVD diamond micro milling cutter with a cutting edge radius of 1.957 μm was also obtained [24].

At present, very few mature investigations are efficiently applied in preparing the required PCD micromilling cutters with large-aspect-ratio, which hinders the development of micromilling technology in machining the complex microstructures. Moreover, inferior surface quality and low machining efficiency seriously restrict the application of deep-and-narrow micromilled grooves in the fields of the aerospace, biomedicine, and space communication.

In this work, a PCD micromilling cutter with a large-aspect-ratio and sharp cutting-edge was firstly fabricated by the developed hybrid method. To verify the cutting performance, comparative experiments of micro-grooves were conducted using the LAR carbide and self-manufactured PCD micromilling cutters. The influence of the milling parameters on the cutting forces and specific energy was explored. Furthermore, the surface quality and machined accuracy of the micromilled grooves with an aspect ratio of 2.5 were investigated, and the tool wear was analyzed.

## 2. Fabrication of Large-Aspect-Ratio (LAR) PCD Micromilling Cutter

### 2.1. Design of LAR PCD Micromilling Cutter

Considering the structural characteristics of deep-and-narrow micromilled grooves with small width (less than 0.5 mm) and high aspect ratio (more than 2), the PCD micromilling cutter developed should meet the conditions of the cutter diameter <0.5 mm and aspect ratio >2 at the same time.

**Figure 1:**
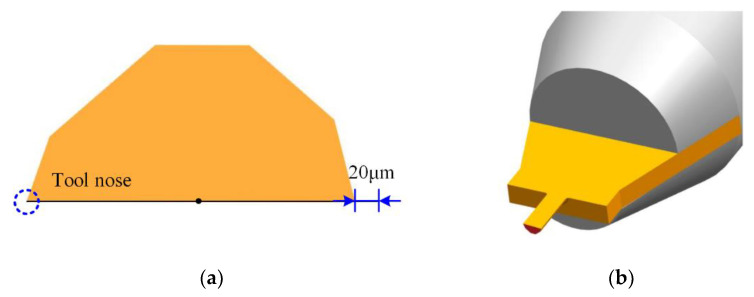
Schematic diagram of designed PCD micromilling cutter. (**a**) Asymmetric hexagon edge structure; (**b**) 3D model.

Preliminary research findings [25,26] have proved that compared with the multi-edged structure, the micromilling cutter with single-edged structure can effectively eliminate the tool-runout size effect, suppress the microburr generation, and improve the machined quality. Furthermore, the single-edged structure is simple, which greatly reduces the tool preparation difficulty. The schematic diagram is given in Figure 1, and the cross-section shape is the asymmetric hexagon. In Figure 1, to avoid the damage to the machined side-walls, the noncutting side was designed to be indented inward by 20 μm. The tool diameter was 0.4 mm, the rake angle and the side-edge clearance angle were 0° and 20°, respectively. The tool holder was made of commercial cemented carbide material, and the holder diameter was 4 mm. The geometric parameters designed are shown in Table 1.

### 2.2. Design of LAR PCD Micromilling Cutter

The primary difficulties in the preparation of an LAR PCD micromilling cutter are summarized as follows: (1) it is difficult to process, and chipping is easy to occur during grinding due to the high hardness of PCD diamond (8–12 times that of carbide material); (2) the small cutter diameter and the large-aspect-ratio reduce the overall stiffness and strength greatly, and the sharp edge is difficult to form unless the grinding force can be strictly controlled; (3) it is easy to produce defects with the increase in grinding length and grinding time for the long side-edge, resulting in inferior quality of the cutting-edge; and (4) high cutting performance requirements, such as sharp edge and wear-resistance. In this work, based on the characteristics of PCD material and existing problems in preparation process, a more efficient hybrid fabrication method combining the laser and grinding is proposed.

**Figure 2:**
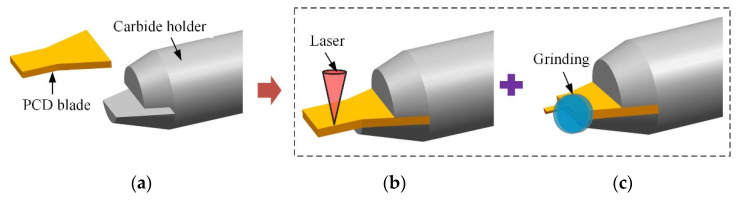
Fabrication scheme of LAR PCD cutter by the hybrid method. (**a**) Blank welding; (**b**) Laser preforming; (**c**) Precision grinding.

The overall scheme was mainly composed of three steps, as shown in Figure 2. Firstly, a customized PCD plate with the size of 2.0 mm × 2.0 mm × 0.4 mm was fixed on a tool holder by the high-frequency induction welding process. Then, the preforming of PCD cutters (including the thinning of the blank, the treatment of the rake face, and preprofiling machining of the edge structures) was realized by laser technology. Finally, the laser-treated PCD cutter was further ground to achieve sharp cutting-edges and satisfactory surface quality.

In general, when preparing a tool with the grinding technology, the traditional steps are firstly rough forming by EDM or laser and then precision grinding. The advantage is that the tool blank only needs to be clamped once in each processing stage, which can effectively avoid the machining error caused by multiple clamping. However, the drawback is the cutter head strength is greatly weakened after the preforming, and a fracture can easily occur in the subsequent grinding due to the influence of the grinding force. Once the blade is broken, the whole tool blank is scrapped, which causes a great waste of blanks and inefficiency of the preparation. Considering above shortcomings and the structural characteristics of the developed LAR PCD cutters, an improved technological process of the alternate mode of the laser forming and precision grinding was proposed, and more details can be found in Ref. [27]. This alternative mode could effectively avoid tool breakage and reduce machining defects. Most importantly, the machined quality of LAR PCD cutters is significantly improved. For the preparation process, the laser parameters used were laser power *P* = 15 W, scanning speed *V* = 0.5 mm/s, and defocusing amount *D_a_* = 0 μm [27]. The grinding conditions utilized were the wheel speed of 19.63 m/s and the workpiece infeed speed of 0.25 mm/min [28].

**Figure 3:**
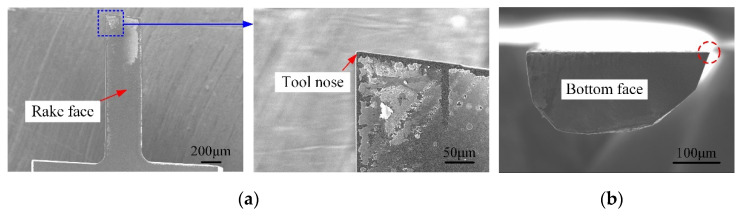
Self-fabricated PCD micromilling cutter. (**a**) Rake face; (**b**) Bottom face.

**Figure 4:**
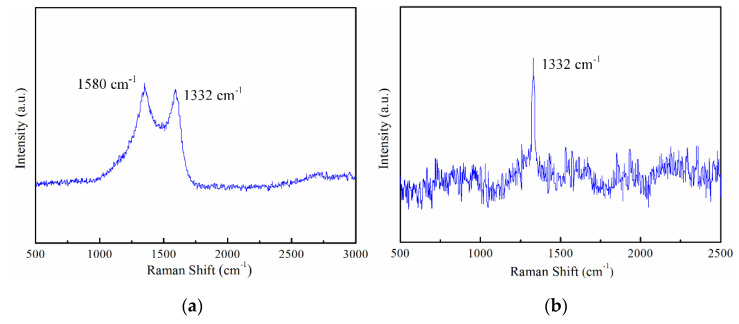
Raman spectra of tool surfaces before and after grinding. (**a**) Before grinding; (**b**) After grinding.

The images of the LAR PCD cutter achieved by scanning electron microscope (Hitachi S3400N, Tokyo, Japan) are displayed in Figure 3. The actual effective diameter and the effective cutting-edge length were about 0.408 mm and 1.3 mm. The aspect ratio obtained was 3.25, which was capable of machining the micro-grooves with a certain depth-to-width ratio. Through careful observation, it was found that the tool nose in Figure 3 is very intact, and the overall quality of the cutting-edges is satisfactory without visible defects. Using a 3D Measurement Laser Microscope (LSM 700, Heidenheim, Germany), the cutting-edge radius and tool nose radius was measured to approximately 2.5 μm and 2.5 μm, respectively. Compared with the EDM-Grinding (cutting-edge radius is about 4.5–5 μm), the cutting-edge quality and tool preparation efficiency of the laser-grinding hybrid technology was significantly improved. Moreover, to further assure the machined quality of the self-fabricated PCD cutter, the phase composition of the flank face before and after the grinding was analyzed by Raman Spectrometer (Renishaw in via, England), and the images are displayed in Figure 4. As can be seen from Figure 4a, a very significant graphite characteristic peak of 1580 cm^−1^ was formed on the laser-formed flank face. However, the graphite characteristic peak completely disappeared after the grinding process with only one Raman characteristic peak locating at 1332 cm^−1^ (Figure 4b), which indicated the thermal effects of the laser on the PCD micromilling cutter were basically eliminated, and no residual graphite or nondiamond phase on the ground surfaces and cutting-edges remained.

## 3. Experimental Setup and Procedures

**Figure 5:**
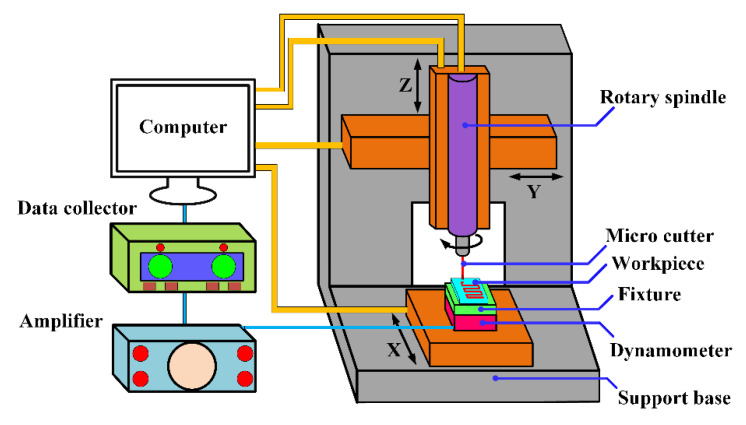
Schematic illustration of the machine device.

Micromilling experiments were carried out on a multifunction high-precision machine, as shown in Figure 5. The platform mainly included a marble structure bed, PMAC control system, air floating high-speed motorized spindle, etc. The maximum spindle speed and rotation accuracy were about 100,000 rpm and 1 μm, respectively. The CCD microscope system was equipped to realize accurate tool setting and realtime monitoring of the machining process. The micro dynamometer (Kistler^®^ 9256C1) was fixed on the lower side of the fixture system. The minimum force measuring threshold and the maximum sampling frequency were 0.002 N and 30 kHz, respectively. The milling force signal was saved by NI-DAQ software after the amplifier system.

**Figure 6:**
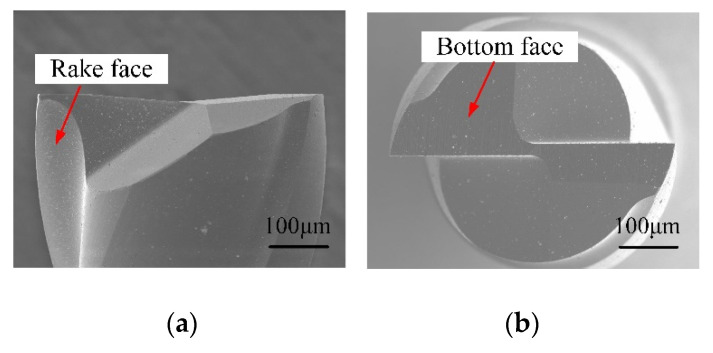
SEM images of carbide micromilling cutter. (**a**) Rake face; (**b**) Bottom face.

The workpiece material employed was No.1 oxygen-free copper (OFC-TU1) with a size of 30 mm × 10 mm × 5 mm, and the main physical properties are provided in Table 2. The self-manufactured LAR PCD cutter was employed, and commercially available two-fluted carbide cutter of 0.5 mm in diameter was included in the comparative experiments, as shown in Figure 6. The carbide cutter was coated with CrTiAlN and the coating hardness was about 3600 HV. The cutter parameters are given in Table 3.

The first part of the experiments was to explore the changes of cutting forces and specific energy, and the scheme is shown in Table 4. The second was the machining of the 1.0-mm-depth micromilled grooves (Table 5), and surface quality, machined accuracy, and tool wear were compared and analyzed. A Hitachi Scanning Electron Microscope (SEM) (S3400N, Tokyo, Japan) was used to observe the surface morphology, microburr formation, and tool wear. The areal surface roughness (*S_a_*) was measured by a 3D confocal microscope (OLS500, Tokyo, Japan).

## 4. Results and Discussion

### 4.1. Cutting Forces and Specific Energy

**Figure 7:**
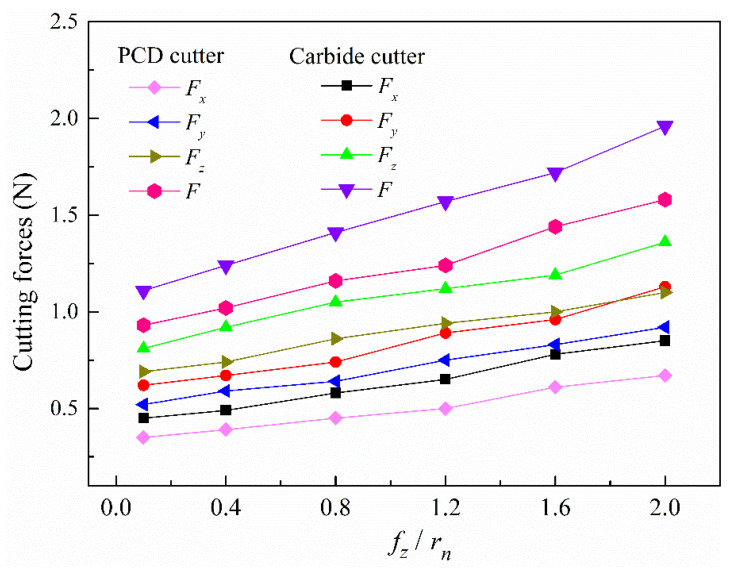
Variations of cutting forces with *f_z_*/*r_n_*.

This section aims to explore the variations of the forces and specific energy with the increase in feed rate/cutting-edge radius (*f_z_*/*r_n_*) with two kinds of micro cutters. The cutting force is one of the important physical characteristics in the micromilling process, which is closely related to chip formation, tool life, and machining status [29]. The forces are recorded in the micromilling process, and the average peak values are taken as the ultimate outcomes. Figure 7 shows the comprehensive response of milling forces for the carbide and self-manufactured PCD cutters.

It can be seen from Figure 7 that the cutting force *F_x_*, *F_y_*, and *F_z_* in X, Y, and Z directions, and the total milling force (*F*) of PCD and carbide micromilling cutters showed a nonlinear growth trend with the increase in *f_z_*/*r_n_*. It should be noted that the force *F_x_*, *F_y_*, and *F_z_* actually represented the terminology of the feed force (*F_f_*), the cross feed force (*F_p_*), and the main force (*F_c_*), respectively. In addition, *F_x_*, *F_y_*, and *F_z_* could be directly measured by the dynamometer. However, by comparison, the corresponding micromilling components and resultant forces of the self-manufactured PCD cutter were smaller than that of the carbide cutter. When *f_z_*/*r_n_* = 0.1, the resultant forces of the carbide and self-manufactured PCD cutters are 1.11 N and 0.93 N, respectively. When *f_z_*/*r_n_* increases to 2, the corresponding resultant forces were 1.96 N and1.58 N, respectively. The force growth of the carbide cutter (0.65 N) was significantly higher than that of self-manufactured PCD cutter (0.85 N). It should be pointed out that the cutting forces of PCD cutter are not so small since the *f_z_*/*r_n_* is only half that of the carbide cutter. The main reason is that from the perspective of metal cutting theory, when the feed rate is doubled, the main cutting force is only increased by about 70–80%, which is not proportional to the feed rate.

**Figure 8:**
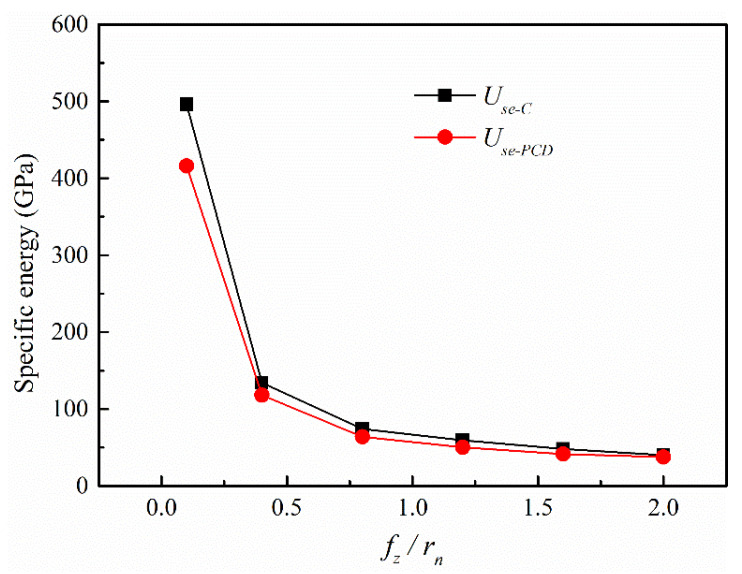
Variation of specific energy with varying *f_z_*/*r_n_*.

Specific energy refers to the required energy to remove a unit volume of the machined material, which is used to characterize the difficulty of the material removal process. For orthogonal cutting, the specific energy (*U_se_*) use can be obtained by the ratio of cutting force to cutting area, as follows:

(1) $$U_{\mathrm{se}}=\frac{F_{\mathrm{cc}}}{S_{c}}=\frac{F_{\mathrm{cc}}}{h_{i}\times d_{w}}\;$$

where *h_i_* and *d_w_* represent the cutting thickness and width, respectively; *F_cc_* represents the force in the cutting speed direction, that is the tangential force (*F_t_*). The tangential force (*F_t_*) and radial force (*F_r_*) can be calculated by the vector decomposition of Equation (2) (where *φ* is the instantaneous contact angle of the cutting tool). According to Equations (1) and (2), the variations of the specific energy under different *f_z_*/*r_n_* conditions can be calculated, and the results are given in Figure 8.

(2) $$\left\{\begin{array}{c} F_{t}=F_{x}\cos\varphi -F_{y}\sin\varphi \\ F_{r}=F_{x}\sin\varphi +F_{y}\cos\varphi \end{array}\right.\;$$

As Figure 8 shows, the specific energy curve of the self-manufactured PCD cutter (*U_se-PCD_*) was located under that of the carbide cutter (*U_se-C_*). This result meant that compared with the carbide cutter, the self-manufactured PCD cutter needed less energy to remove a unit volume of the OFC-TU1 material, and the material removal process was relatively easier. Meanwhile, with the decrease in *f_z_*/*r_n_*, both *U_se-PCD_* and *U_se-C_* increased nonlinearly, and the smaller the *f_z_*/*r_n_* was, the more significant the nonlinear characteristics were.

In micromilling, the size effect becomes very serious when the *f_z_*/*r_n_* is small to a certain extent, and the specific energy increases sharply as the *f_z_*/*r_n_* decreases further. What follows is that more energy is required to remove unit volume material, and the chip formation becomes more difficult. The reason is that the contact area between the cutting-edge and the workpiece material increases with the decrease in *f_z_*/*r_n_*, which leads to a larger friction force. When the friction force exceeds the shear force that is required to shear the material into chips, the specific energy raises sharply, and the processed material flows to both sides and downside of the cutter (that is plastic deformation) [30]. At this moment, there is no chip outflow from the rake face. With the continuous rotation of the cutter teeth, the workpiece material gradually accumulates, and the instantaneous cutting thickness begins to increase. When the instantaneous cutting thickness approaches the minimum cutting thickness, chips begin to form. As the *f_z_*/*r_n_* continues to grow, the size effect becomes weaker, and the proportion of the cutting-edge arc participating in material removal process is in decline. The shear effect gradually becomes the dominant machining mechanism, and the material removal process is easier and easier [31]. In addition, the self-manufactured PCD cutter had a larger chip removal space, which provided more favorable conditions for strip chip formation and outflow. This is also an important reason why the material removal and chip formation of the self-manufactured PCD cutter was smoother than that of the carbide cutter under the same conditions.

### 4.2. Surface Quality of Side-Walls and Machined Accuracy

**Figure 9:**
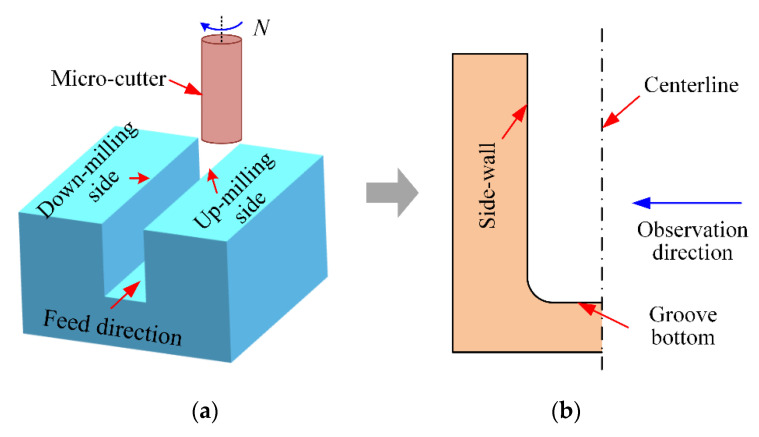
Observation diagram of side-walls. (**a**) HAR micro-groove; (**b**) Observation direction.

**Figure 10:**
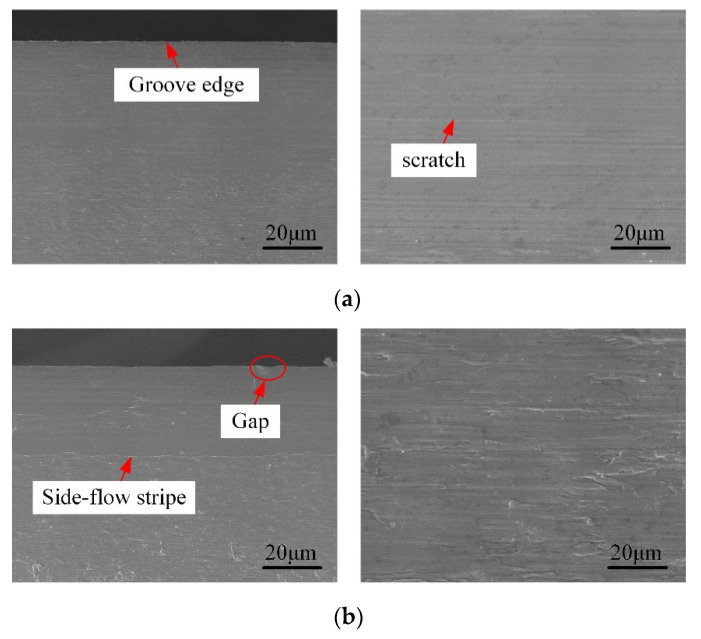
Surface morphology of side-walls of deep-and-narrow micromilled grooves. (**a**) Self-manufactured PCD cutter; (**b**) Carbide cutter.

The surface quality of side-walls is critical for the causative performance of micromilled grooves [32]. Firstly, in order to observe the machined quality of side-walls conveniently, the micromilled grooves obtained were longitudinally cut along the center line of the groove bottom by wire electrical discharge machining (WEDM). The observation diagram is shown in Figure 9. Then the surface morphology and areal surface roughness were measured by SEM (S3400N, Tokyo, Japan) and 3D confocal microscope (OLS500, Tokyo, Japan). Figure 10 displays the SEM results of the side-wall surface morphology in the up-milling side achieved by the carbide cutter and the self-manufactured PCD cutter.

**Figure 11:**
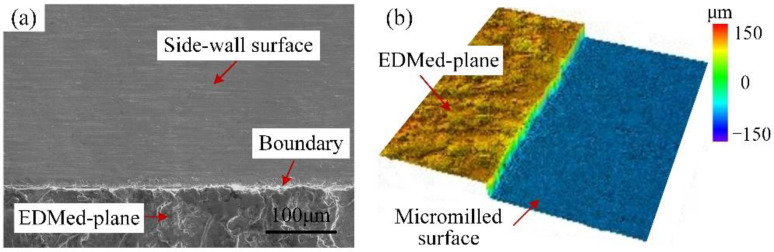
Surface morphology of the bottom boundary of side-walls by carbide cutter. (**a**) 2D morphology; (**b**) 3D morphology.

As can be seen from Figure 10b, the side-wall surface was mainly characterized by a fuzzy texture and indistinct tool marks. The uneven tool nose scratches were visible, indicating the material plastic side-flow was extremely serious; meanwhile, some large microgaps were discovered on the upper edge of the milled groove. In Figure 10b, a very prominent and nearly white thin side-flow stripe is clearly formed at about 300 μm away from the upper edge, and the side-wall surface was further divided into the upper region and lower region. The upper region was relatively regular with few defects, whereas the surface defects in the lower area became more severe along the direction near the bottom edge of the milled groove. In addition, Figure 11 reveals that the closer to the bottom edge, the more serious chip adhesion and covering phenomenon there was on the side-wall surface. In Figure 11b, the micromilled surface and the EDMed-plane were connected by a transition area with a certain width, which was almost completely covered by accumulated or overlaid material. At this time, it was difficult to distinguish the boundary between the side-wall surface and the EDMed-plane clearly. The primary reason for the above results is the severe tool wear of the carbide cutter. Before the milling distance reached 1200 mm (the groove depth is 1.0 mm), the carbide cutter almost had worn out, and the cutting-edge sharpness was completely lost. The interaction effect between the cutting-edge and workpiece material was very insufficient; meanwhile, the uneven wear was more serious due to the two-fluted carbide cutter caused by the spindle radial runout. All these factors aggravated the lamination and accumulation of chips.

**Figure 12:**
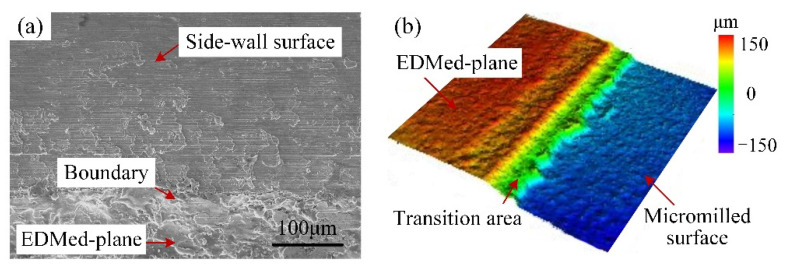
Surface morphology of the bottom boundary of side-walls by the self-fabricated PCD cutter. (**a**) 2D morphology; (**b**) 3D morphology.

For the self-manufactured PCD cutter, it can be seen from Figure 10a that the side-wall surface quality was satisfactory with few scratches and clear tool-marks, and the material lamination and adhesion were also relatively slight. Though the machining defects (like microburrs and scratched) also became more prominent from the top edge to the groove bottom, the overall upper side-wall surface obtained by the self-manufactured PCD cutter was relatively flatter compared to that of the carbide cutter in Figure 10b. As shown in Figure 12, there was only slight material accumulation and lamination at the bottom of the side-wall, and the distribution area was also limited; meanwhile, the boundary between the micromilled surface and the EDMed-plane was clearly visible. Furthermore, the average areal surface roughness of the side-walls obtained from the PCD and carbide cutters was measured to be 159 nm and 305 nm, respectively, and the former was obviously smaller than the latter. Therefore, all these results fully indicate that the self-manufactured LAR PCD cutter kept a sharp edge for a longer time under the same conditions and milling distance, and had strong sustainable processing ability.

**Figure 13:**
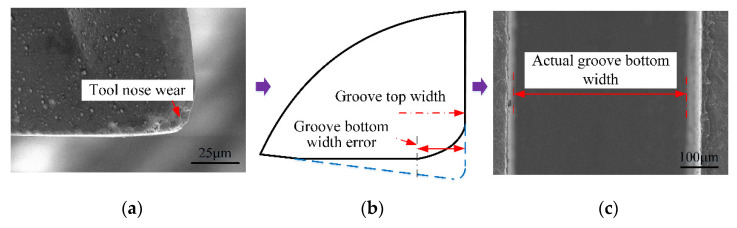
Worn out tool nose shape of carbide micromilling cutter and its influence on micro-groove size.

When the carbide micromilling cutter was worn, the coating material in the triangular three-dimensional curved surface area near the tool nose was gradually ground off with the continuous machining process (Figure 13a). After a large number of coatings fell off, the cemented carbide matrix had difficulty performing the cutting work, and then it was quickly worn out, resulting in the increase in the tool nose radius and the cutting-edge radius. In addition, the effective diameter decreased with the large tool nose radius, which the machined error of the groove bottom (Figure 13b). At the same time, the geometry of the micromilling cutter changed suddenly, and the integrity of the cutting edge was seriously damaged. The actual groove bottom width obtained was greatly reduced, as shown in Figure 13c.

**Figure 14:**
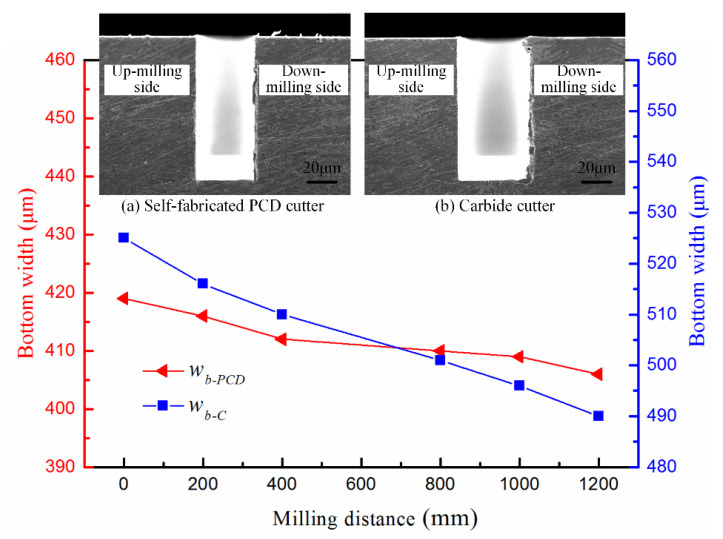
Variation of the bottom widths (*w_b_*) with the milling distance.

Furthermore, Figure 14 shows the variation of the width of the groove bottom (*w_b_*) with the milling distance for the self-manufactured PCD and commercial carbide cutters. It can be seen that when the milling distance reached 1200 mm (the groove depth was 1.0 mm), the groove width of the top and bottom processed by the carbide cutter (the original diameter was 0.5 mm) was 525 μm and 496 μm respectively, and the difference was 29 μm. For the self-manufactured PCD cutter, they were 414 μm and 406 μm, respectively, and the difference was 8 μm. By comparison, the size error of the micromilled groove obtained by the self-manufactured PCD cutter was much smaller than that of carbide cutter. The primary reason is the different wear degree of these two kinds of cutters, which can be further verified from the tool wear results in Section 4.3. With the increase in the milling distance and groove depth, the carbide cutter wore out rapidly, resulting in a larger cutting-edge radius and tool nose radius, as well as a smaller effective diameter. The integrity of the cutting-edge and the geometry of the tool nose were seriously damaged. The surface generation of the micromilled groove was the instantaneous reflection of the tool nose geometry in the cross-sectional profile direction, and the groove width was extremely sensitive to the change of the tool nose radius. The instantaneous groove width varied with the tool nose radius, resulting in the size error in different depths of micromilled grooves. The wear-resistance and sustainable processing ability of the self-manufactured PCD cutter was stronger, and the machining error was also smaller.

### 4.3. Tool Wear

**Figure 15:**
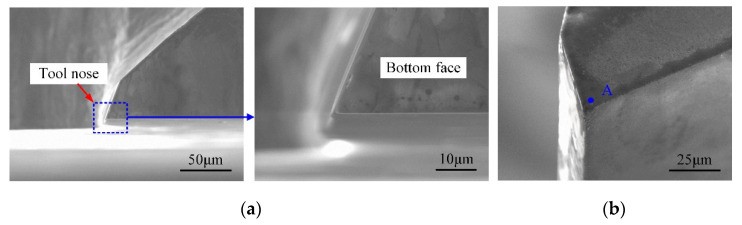
Tool wear of the self-fabricated PCD micromilling cutter. (**a**) Top view of bottom surface; (**b**) Tool nose area.

**Figure 16:**
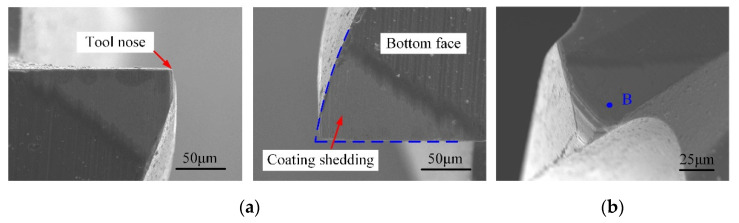
Tool wear of two-fluted carbide micromilling cutter. (**a**) Top view of bottom surface; (**b**) Tool nose area.

**Figure 17:**
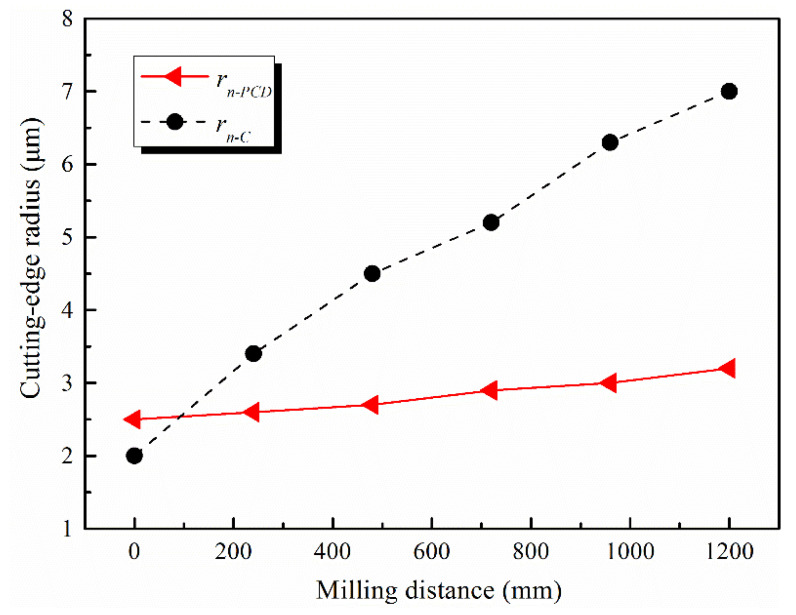
Variations of cutting-edge radii with milling distance.

**Figure 18:**
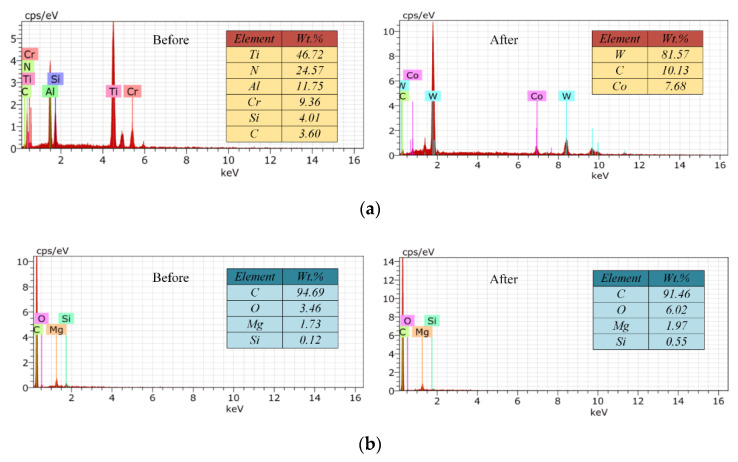
EDS results before and after machining a 1.0-mm-depth groove. (**a**) Carbide cutter; (**b**) Self-manufactured PCD cutter.

The tool wear of the self-manufactured PCD and commercial carbide cutter after cutting 1.0-mm-depth grooves are illustrated in Figure 15 and Figure 16, and the total milling distance of each cutter was 1200 mm. Figure 17 shows the variation of the cutting-edge radius (*r_n-PCD_* and *r_n-C_*) with the increase in the milling distance. Furthermore, EDS spectra of the tool noses before and after machining 1.0-mm-depth groove were analyzed, and the results are shown in Figure 18.

For the carbide cutter in Figure 16, the wear was very severe with a large amount of coating material shedding near the tool nose, and the initial geometry of sharp cutting-edge no longer existed. In general, the tool wear developed gradually with the cutting time, and mainly went through three stages of initial wear, normal wear, and severe wear. Combined with Figure 17, for the same milling distance, the variation curve of *r_n-C_* was always in the rapid growth mode, while the *r_n-PCD_* curve was relatively gentle. When the milling distance reached 1200 mm, *r_n-C_* varied from 2 μm to 7 μm, and the growth rate was 225%. In micromilling, the spindle radial runout and the symmetry manufacturing deviation of micro-cutters with multi-edges are equivalent to the milling parameters, which makes the micromilling process extremely sensitive, further resulting in the difference of the instantaneous cutting thickness of each tooth. Only one cutting-edge is involved in cutting in some cases, and the others are in a noneffective cutting state (e.g., extrusion and ploughing). Hence, a few cutting-edges would enter the severe wear stage prematurely [33]. As shown in Figure 16, the wear degree of the two teeth of the carbide cutter was not consistent, and the uneven milling phenomenon was prominent.

In addition, the main significance of the coating treatment of carbide cutters is to combine the basic characteristics of tool substrate material and coating material, so as to improve the comprehensive performance of carbide cutters. However, the intermittent impact load during the micromilling process accelerated the coating shedding and even caused the chipping of the tool nose (Figure 16a). When the coating material in the cutting area was completely peeled off, the wear and failure of carbide cutter became faster and more severe. In Figure 18a, EDS analysis (point B in Figure 16b) showed that in the worn area the constituent elements of coating material CrTiAlN disappeared, and the W, Co, and C became the main elements. This indicated that after machining for a 1200 mm distance, a large number of the coating materials fell off the tool nose area, resulting in the wear-resistance and sharpness of carbide cutter no longer existing.

For the self-manufactured PCD cutter, the wear degree of the cutting-edge and tool nose area was relatively slight (Figure 15). Figure 17 reveals the variation of *r_n-PCD_* was very small, and the increased amplitude was estimated at about 28%. This illustrates that the self-manufactured PCD cutter kept higher sharpness when the milling distance reached 1200 mm. EDS results of point A in Figure 15b show that the material composition of PCD diamond basically had no change (Figure 18b), which revealed once again that the self-manufactured PCD cutter had strong wear-resistance and continuous processing ability. Moreover, the single-edged structure of the PCD cutter completely avoided the uneven wear phenomenon in micromilling. Therefore, compared with the two-edged coated carbide micromilling cutter, under the same milling parameters and milling distance, the self-made large-aspect-ratio PCD cutter had obvious advantages in wear-resistance, continuous processing ability, and service life.

Furthermore, what should be highlighted is that although the higher performance and sharper diamond micromilling cutters are constantly fabricated by advancing technology, the depth-to-width ratio of the micro-grooves with micromilling technology is limited by the aspect ratio obtained by the micromilling cutters. The results suggest that the more precise geometric structure of diamond micromilling cutters should be designed to improve the surface quality of the high-aspect-ratio microstructures, and more reliable fabrication technology needs to be further developed to obtain larger aspect ratio diamond micromilling cutters.

## 5. Conclusions

In this paper, systematic research into deep-and-narrow micromilled grooves by a self-manufactured PCD cutter was carried out. Firstly, a large-aspect-ratio PCD cutter was fabricated by the developed hybrid method of laser and grinding. Then, micromilling experiments of oxygen-free copper material were conducted to investigate the machined surface quality of the micro-grooves. The primary conclusions obtained from the results are as follows:

(1) A superior quality PCD cutter was successfully manufactured by the proposed hybrid method of laser and grinding. The aspect ratio and cutting-edge radius achieved were 3.25 and 2.5 μm.
(2) In comparison, under the same conditions, both the milling forces and specific energy corresponding to the self-manufactured single-edged PCD cutter were smaller than that of the commercial two-fluted carbide cutter. The material removal process was easier for the self-manufactured PCD cutter.
(3) A satisfactory micromilled groove with a depth-to-width ratio of 2.5 was achieved by using the self-fabricated LAR PCD cutter under the optimized conditions. The side-wall surfaces were smoother and flatter with few defects, and the geometric dimension error of the obtained micro-groove was much smaller (8 μm).
(4) When the milling distance reached 1200 mm, the tool wear of the self-manufactured LAR PCD cutter was relatively slight. The increased amplitude of the cutting-edge radius was about 28%, only normal wear has occurred.

The self-manufactured PCD micromilling cutter with a large-aspect-ratio provided an efficient technology program for the preparation of micro-grooves with high depth-to-width ratio and small scale. This work is further expected to be of positive significance to improve the machined quality and efficiency of the micromilled grooves. Future research should concentrate on the fabrication of diamond micro cutters with more stable cutting performance.

## Figures and Tables

**Table 1:** Geometric parameters of LAR PCD micromilling cutter.

Parameters	Cutter
Flute number	1
Grain size	10
Total length (mm)	45
Holder diameter (mm)	4
Edge diameter (mm)	0.4
Rake angle (°)	0
Inclination angle (°)	5
Blank edge length (mm)	2

**Table 2:** Physical properties of OFC-TU1 material.

Properties	Values
Specific heat J/(kg·°C)	385
Thermal conductivity W/(m·°C)	391
Young’s modulus (GPa)	124
Shear Modulus (GPa)	47.7
Poisson ratio	0.34
Density (kg/m^3^)	8.96 × 103

**Table 3:** Geometric parameters of LAR PCD and carbide cutters.

Parameters	PCD	Carbide
Flute	1	2
Diameter (mm)	0.4	0.5
Effective edge length (mm)	1.3	1.5
Aspect ratio	3.25	3
Cutting-edge radius *r_n_* (μm)	2.5	2
Tool nose radius *r_ɛ_* (μm)	2.5	2
Coating material	-	CrTiAlN

**Table 4:** Details of the parameter experiments.

Parameters	Values
Spindle speed *n* (rpm)	20,000
Depth of cut *a_p_* (μm)	5
Feed rate *f_z_* (μm/z)	0.25, 0.5, 1, 2, 3, 4, 5, 6
Workpiece material	OFC-TU1
Cutting fluid	Water-free alcohol

**Table 5:** Parameters of deep-and-narrow micromilled grooves.

Cutters	*n* (rpm)	*a_p_* (μm)	*f_z_*/*r_n_*	Depth (mm)	Length (mm)
PCD	20,000	5	0.4	1.0	6.0
Carbide	20,000	5	0.8	1.0	6.0

## Nomenclature

*P*	laser power (W)
*V*	scanning speed (mm/s)
*D_a_*	defocusing amount (μm)
*r_n_*	cutting-edge radius (μm)
*r_n-PCD_*, *r_n-C_*	cutting-edge radius of PCD and carbide cutter (μm)
*r* *_ɛ_*	Tool nose radius (μm)
*n*	Spindle speed (rpm)
*a_p_*	depth of cut (μm)
*f_z_*	Feed rate (μm/z)
*f_z_/r_n_*	feed rate/cutting-edge radius
*S_a_*	areal surface roughness (μm)
*F_x_, F_y_, F_z_*	cutting forces in X, Y, and Z directions (N)
*F_f_, F_p_, F_c_*	cutting forces of feed, cross feed, and main force (N)
*F_t_, F_r_*	cutting forces in tangential and radial directions (N)
*F_cc_*	force in the cutting speed direction (N)
*U_se_*	specific energy (GPa)
*U_se-PCD_*, *U_se-C_*	specific energy of PCD and carbide cutter (GPa)
*h_i_, d_w_*	cutting thickness and width (μm)
*φ*	instantaneous contact angle (°)

## References

[1] Bhardwaj R.K., Sudhamani H.S., Dutta V.P., Bhatnagar N. (2021). Micromachining and Characterisation of Folded Waveguide Structure at 0.22 THz. J. Infrared Millim. Terahertz Waves.

[2] Babaeihaselghobi A., Ghavifekr H.B. (2020). Development of a planar helix slow-wave structure based on ball-grid array technology. J. Electromagn. Waves Appl..

[3] Dong H.N., Ahn H.S. (2021). A comprehensive review on micro/nanoscale surface modification techniques for heat transfer enhancement in heat exchanger. Int. J. Heat Mass Transf..

[4] Wang Y.S., Zou B., Huang C.Z., Liu Z.Q., Yao P. (2018). The micro-cutting performance of cermet and coated WC micro-mills in machining of TC4 alloy micro-grooves. Int. J. Adv. Manuf. Technol..

[5] Hu P., Lei W.Q., Jiang Y., Huang Y.H., Song R., Chen H.B., Dong Y. (2018). Development of a 0.32-THz Folded Waveguide Traveling Wave Tube. IEEE Trans. Electron Devices.

[6] Han J.J., Hao X.Q., Li L., Liu L.H., Chen N., Zhao G.L., He N. (2020). Investigation on Surface Quality and Burr Generation of High Aspect Ratio (HAR) Micro-Milled Grooves. J. Manuf. Process..

[7] Bang Y.B., Lee K.M., Oh S. (2005). 5-axis micro milling machine for machining micro parts. Int. J. Adv. Manuf. Technol..

[8] Llanos I., Agirre A., Urreta H., Thepsonthi T., Özel T. (2014). Micromilling high aspect ratio features using tungsten carbide tools. Proc. Inst. Mech. Eng. Part B J. Eng. Manuf..

[9] Kou D.J., Wan Y., Liu Z.Q., Cai Y.K., Liang X.C. (2015). Deformation control in micro-milling of thin-walled structures. Int. J. Adv. Manuf. Technol..

[10] Zariatin D.L., Kiswanto G., Ko T.J. (2017). Investigation of the micro-milling process of thin-wall features of aluminum alloy 1100. Int. J. Adv. Manuf. Technol..

[11] Malayath G., Sidpara A.M., Deb S. (2020). Study of different materials response in micro milling using four edged micro end mill tools. J. Manuf. Process..

[12] Niu Z., Jiao F., Cheng K. (2018). An innovative investigation on chip formation mechanisms in micro-milling using natural diamond and tungsten carbide tools. J. Manuf. Process..

[13] Deng B., Peng F.Y., Zhou L., Wang H.W., Yang M.H., Yan R. (2020). A comprehensive study on flank wear progression of polycrystalline diamond micro-tool during micro end-milling of SiCp/Al composites. Wear.

[14] Nakamoto K., Katahira K., Ohmori H., Yamazaki K., Aoyama T. (2012). A study on the quality of micro-machined surfaces on tungsten carbide generated by PCD micro end-milling. CIRP Ann..

[15] Eberle G., Jefimovs K., Wegener K. (2015). Characterisation of thermal influences after laser processing polycrystalline diamond composites using long to ultrashort pulse durations. Precis. Eng..

[16] Liang Z.Q., Li S.D., Zhou T.F., Wang X.B., Gao P., Zhang D.D., Teng L.L. (2018). Fabrication and milling performance of micro ball-end mills with different relief angles. Int. J. Adv. Manuf. Technol..

[17] Yuan H., Zhao W.X., Liang Z.Q., Li S.D., Wang X.B., Zhou T.F., Sun X.F., Jiang L.K. (2020). Structural design and fabrication of polycrystalline diamond micro ball-end mill. Int. J. Adv. Manuf. Technol..

[18] Zhang Z.Y., Peng H.M., Yan J.W. (2013). Micro-cutting characteristics of EDM fabricated high-precision polycrystalline diamond tools. Int. J. Mach. Tool. Manu..

[19] Chen N., Li H.N., Wu J.M., Li Z.J., Li L., Liu G.Y., He N. (2021). Advances in micro milling: From tool fabrication to process outcomes. Int. J. Mach. Tool. Manu..

[20] Suzuki H., Moriwaki T., Yamamoto Y., Goto Y. (2007). Precision cutting of aspherical ceramic molds with micro PCD milling tool. CIRP Ann..

[21] Oliaei S.N.B., Karpat Y. (2018). Polycrystalline diamond end mill cutting edge design to improve ductile-mode machining of silicon. Precis. Eng..

[22] Ogawa Y., Ota M., Nakamoto K., Fukaya T., Russell M., Zohdi T.I., Yamazaki K., Aoyama H. (2016). A study on machining of binder-less polycrystalline diamond by femtosecond pulsed laser for fabrication of micro milling tools. CIRP Ann..

[23] Jiang J.L., Sun S.F., Wang D.X., Yang Y., Liu X.F. (2020). Surface texture formation mechanism based on the ultrasonic vibration-assisted grinding process. Int. J. Mach. Tools Manuf..

[24] Yang K., Xia Y., Li L., He N., Zhang Y., Zhang T., Wang Y. (2018). Experimental study on hybrid machining of laser irradiation and grinding for sharpening of a CVD diamond micro-milling tool. Int. J. Adv. Manuf. Technol..

[25] Zhan Z.B., He N., Li L., Shrestha R., Liu J.Y., Wang S.L. (2015). Precision milling of tungsten carbide with micro PCD milling tool. Int. J. Adv. Manuf. Technol..

[26] Wu X., Li L., He N., Zhao G.L., Jiang F. (2019). Fabrication of PCD micro end mill for machining hard and brittle material. Int. J. Adv. Manuf. Technol..

[27] Han J.J., Hao X.Q., Li L., He N., Zhao G.L., Chen N. (2019). Fabrication of large aspect ratio (LAR) PCD micro-end mill with a hybrid method and performance verification. Int. J. Adv. Manuf. Technol..

[28] Zhan Z.B., Li L., He N., Shrestha R. (2014). An experimental study on grinding parameters for manufacturing PCD micro-milling tool. Int. J. Adv. Manuf. Technol..

[29] Jing X.B., Lv R., Chen Y., Tian Y.L., Li H.Z. (2020). Modelling and experimental analysis of the effects of run out, minimum chip thickness and elastic recovery on the cutting force in micro-end-milling. Int. J. Mech. Sci..

[30] Chen N., Li L., Wu J.M., Qian J., He N., Reynaerts D. (2019). Research on the ploughing force in micro milling of soft-brittle crystals. Int. J. Mech. Sci..

[31] Chen W.Q., Huo D.H., Teng X.Y., Sun Y.Z. (2017). Surface Generation Modelling for Micro end Milling Considering the Minimum Chip Thickness and Tool Runout. Procedia CIRP.

[32] Hao X.Q., Xu W.H., Chen M.Y., Wang C., Han J.J., Li L., He N. (2021). Laser hybridizing with micro-milling for fabrication of high aspect ratio micro-groove on oxygen-free copper. Precis. Eng..

[33] Singh K.K., Kartik V., Singh R. (2019). Stability Modeling with Dynamic Run-out in High Speed Micromilling of Ti6Al4V. Int. J. Mech. Sci..

